# First person – Miguel Ramirez-Moreno

**DOI:** 10.1242/dmm.052871

**Published:** 2026-03-05

## Abstract

First Person is a series of interviews with the first authors of a selection of papers published in Disease Models & Mechanisms, helping researchers promote themselves alongside their papers. Miguel Ramirez-Moreno is first author on ‘ *Drosophila* wing is a high-throughput and versatile screening tool for Tau-mediated disease mechanisms and drug discovery’, published in DMM. Miguel is a postdoctoral researcher in the lab of Professor Amritpal Mudher at University of Southampton, Southampton, UK, investigating how life works with the goal of improving everyone's health.


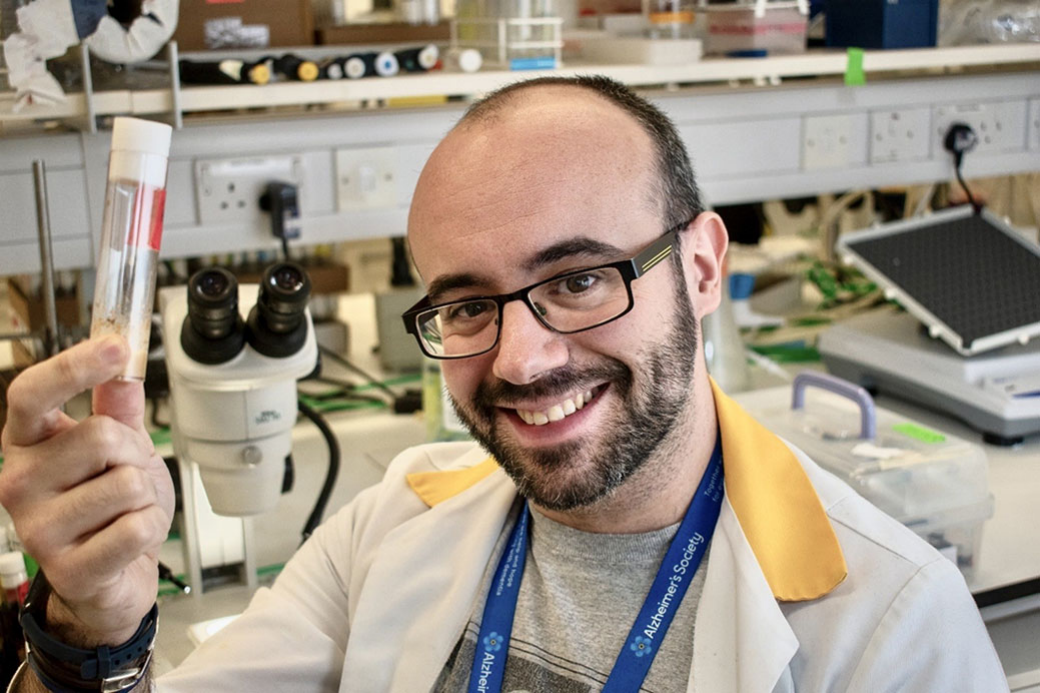


**Miguel Ramirez-Moreno**

**Who or what inspired you to become a scientist?**

I don't have a single person on a pedestal; instead, I carry a whole bag filled with small crumbs of inspiration. Going back to the very beginning, it wouldn't be me if I didn't admit that, after watching a certain film as a child and learning that both dinosaurs and humans are made of DNA, I promised myself that – rather than becoming an astronaut or a firefighter – I would dedicate my life to discovering dinosaurs or even creating them! I should mention, though, that the moment I really saw myself at the bench was during a practical session in my undergraduate degree. Our lecturers – Marta, Jony, Laura and our dearly missed Macu – taught us so much about working with fruit flies during four unforgettable weeks.

**What is the main question or challenge in disease biology you are addressing in this paper? How did you go about investigating your question or challenge?**

The main point of our paper is introducing a new fast and versatile *in vivo* system to study Tau-mediated disease mechanisms and to screen potential therapeutic compounds. Most current tauopathy models rely on ageing nervous systems that take weeks to months to exhibit pathology and are therefore slow, resource intensive and unsuitable for high-throughput screening. In comparison, researchers can now use the *Drosophila* wing disc as a quicker alternative assay with highly scoreable phenotypes in the adult wing and the possibility of investigating cellular pathways in the larval wing disc. We show that expression of human Tau inside the wing disc cells induces quantifiable toxicity, and we proceed to validate critical aspects of Tau pathogenesis and how they impact toxicity. We used custom Tau variants that lack aggregation motifs (VQIVYK and VQIINK) or carry phosphomimetic substitutions (E14) to recapitulate the same responses we previously observed in *Drosophila* neurons. We also confirmed that clinically relevant point mutations in the Tau sequence, like R406W, enhance toxicity in the wing enough to be distinguished from the original protein. To validate the wing as a drug-screening tool, we fed the animals the peptide NAP, which reduced Tau-induced toxicity. Overall, we have established an efficient, scoreable and mechanistically informative *in vivo* screening platform for Tau toxicity.

**How would you explain the main findings of your paper to non-scientific family and friends?**

Tau is a protein that normally helps our neurons talk to each other, but it misbehaves in Alzheimer's disease and other maladies that cause dementia. As part of the effort in finding a cure, we need to understand why Tau protein becomes hurtful to our neurons and how to use drugs to fix the problem. The main idea of our work is that we found a very quick and simple way to study this protein by using the wings of fruit flies, which surprisingly react to Tau in a similar way to human brain cells. However, inspecting the damage in the fly wing is much easier and faster than in the brain, facilitating testing of candidate medicines and therapies. We can also trial different versions of the protein to learn which parts make it dangerous and how to make it less harmful. Our findings show that the fly wing turns out to be a very convenient and low-cost way to explore big questions about Alzheimer's disease. Overall, we brought to the spotlight a fast and powerful tool that can help researchers understand the disease better and speed up the search for new treatments.

Overall, we brought to the spotlight a fast and powerful tool that can help researchers understand [Alzheimer's] disease better and speed up the search for new treatments

**What are the potential implications of these results for disease biology and the possible impact on patients?**

We have recapitulated multiple aspects of Tau pathology using the *Drosophila* wing. This model could accelerate discoveries about why diseases like Alzheimer's arise and how to stop their progression. The ability to pinpoint which parts of the Tau protein make it toxic – using phosphomimetic or clinically relevant mutations – and which changes reduce its toxicity may help identify new targets for future treatments. The platform also allows scientists to quickly test candidate drugs, as shown when the neuroprotective NAP peptide reduced Tau-induced damage. Since this system works in days rather than months, it greatly increases the number of compounds, mutations or other factors that can be screened in a single project. Faster and more efficient preliminary testing means that only the most promising candidates would need to be validated in more clinically relevant models that require more time and resources. While work in flies is only the first step, these findings could eventually contribute to better treatment strategies and, in the long term, improve outcomes for patients.

**Figure:**
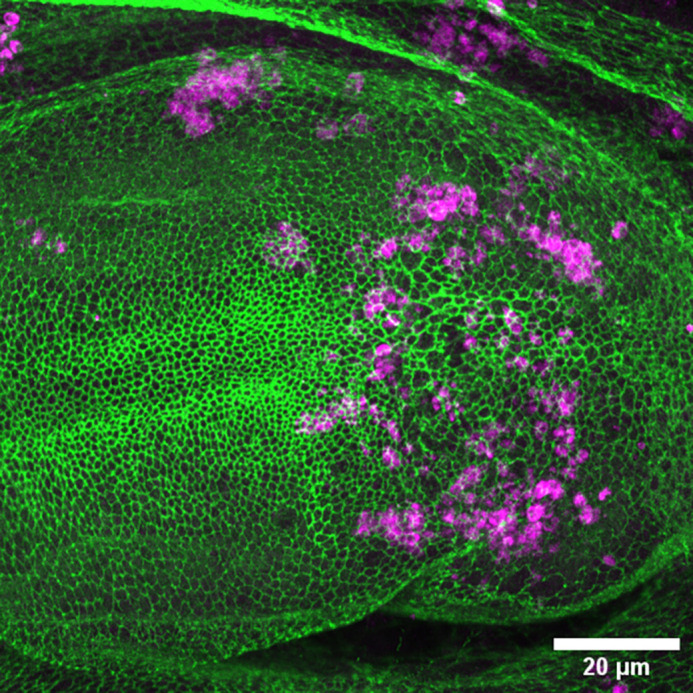
**The first result that led to this publication.** How would the cells that make up the fly wing (green; antibody against the membrane protein E-cadherin) react if I filled them with human Tau protein? Very badly, given the number of cells undergoing apoptosis (magenta; antibody against cleaved Caspase Dcp-1). The difference between the cells that express Tau (right side of the image) and the unaffected ones (left side of the image) can be very striking.

**Why did you choose DMM for your paper?**

I was, of course, already familiar with The Company of Biologists – both as a reader and as a grateful recipient of many of their conference freebies! I also appreciated their openness to publishing *Drosophila*-related research. As the story of our manuscript took shape, demonstrating how several disease mechanisms attributed to the Tau protein are recapitulated in a new paradigm for the field, choosing DMM felt almost self-evident.

**Given your current role, what challenges do you face and what changes could improve the professional lives of other scientists in this role?**

‘Postdoctoral researcher’ is a very vague term, covering everything from after your PhD until securing a tenured academic position. Yet, all share one difficult feature: fixed-term contracts. Regardless of whether someone is happy to remain a postdoc long-term or aspires to become a group leader, their income depends on uncertain contract renewals or unpredictable funding calls. This is very taxing, especially for those trying to build a stable life, move countries or start a family. We urgently need to offer more stability to early-career researchers who want a realistic pathway toward becoming principal investigators. We also need a fairer system to evaluate postdocs for the full range of work they do – work that is often essential for their research communities – but which currently goes unrecognized if it doesn't result in publications.

**What's next for you?**

I want to take full advantage of the model and use it to identify novel factors that either promote pathology relevant to dementia or protect us from it. My goal is to uncover new mechanisms that can move dementia research forward and ultimately contribute to better interventions.

**Tell us something interesting about yourself that wouldn't be on your CV**

Outside of science, writing fiction helps me reconnect with my personal hobbies and my mother tongue. I have published several short stories and have been a finalist in a couple of contests. As a writer, I am also part of a small but very capable studio that released its first video game last year.
